# Paine’s Materia Medica

**Published:** 1848

**Authors:** 


### ARTICLE V.

*Materia Medica and Therapeutics.* By Martin Paine, A.M., M.D., Professor of the Institutes of Medicine and Materia Medica in the University of New York, etc. New York: Samuel S. & William Wood. 1848. 12mo. pp. 411.

This work is what it professes to be—a compendium of therapeutics: giving also a short but comprehensive view of the relative merits of the principal articles of the Materia Medica, arranged in the order of their relative value, with many useful directions for their preparation and combination.

It will be found to be a useful and convenient book for determining readily the properties and doses of different medicines and for ascertaining what are the proper doses and most approved formulæ to be used in their administration, and as such we cheerfully recommend it. H.

